# Tranexamic Acid Reduces Total Blood Loss and Inflammatory Response in Computer-Assisted Navigation Total Knee Arthroplasty

**DOI:** 10.1155/2019/5207517

**Published:** 2019-12-09

**Authors:** Kuan-Ting Wu, Ka-Kit Siu, Jih-Yang Ko, Wen-Yi Chou, Shu-Jui Kuo, Ya-Hung Hsu

**Affiliations:** ^1^Department of Orthopedic Surgery, Kaohsiung Chang Gung Memorial Hospital, Kaohsiung, Taiwan; ^2^Center for Shockwave Medicine and Tissue Engineering, Department of Medical Research, Kaohsiung Chang Gung Memorial Hospital, Kaohsiung, Taiwan; ^3^Core Lab for Phenomics and Diagnostics, Kaohsiung Chang Gung Memorial Hospital, Kaohsiung, Taiwan; ^4^Department of Orthopedic Surgery, Xiamen Chang Gung Hospital, Xiamen, Fujian, China; ^5^Department of Orthopedic Surgery, China Medical University Hospital, Taichung, China

## Abstract

**Introduction:**

Tranexamic acid (TXA) is an effective blood salvage agent that reduces perioperative blood loss in conventional total knee arthroplasty (TKA). As computer-assisted surgery for TKA (CAS-TKA) results in a lower perioperative blood loss than conventional TKA, the additional effect of blood conservation by TXA might be mitigated. This study aimed to evaluate the efficacy of TXA in CAS-TKA.

**Methods:**

We retrospectively reviewed 222 consecutive patients who underwent CAS-TKA. Intravenous TXA was administered in 103 patients (TXA group) at a dosage of 20 mg/kg 15 min before deflation of the tourniquet. The other 119 patients did not receive TXA (control group). Patient demographic data including age, gender, BMI, DM, and hypertension were collected. The primary outcomes were the estimated total blood loss (ETBL) and perioperative data, including tourniquet duration, preoperative and postoperative day 1 (POD1) and day 3 (POD3) serum D-dimer, CRP, hemoglobin (Hb), and hematocrit (Hct) levels. Secondary outcomes including transfusion rate and 90-day complications were recorded.

**Results:**

The ETBL was lower in the TXA group on both POD1 (404.34 ± 234.77 vs. 595.47 ± 279.04, *p* < 0.001) and POD3 (761.39 ± 260.88 vs. 987.79 ± 326.58, *p* < 0.001). The TXA group also demonstrated a lower level of CRP on POD1 (*p*=0.02) and lower levels of CRP and serum D-dimer on POD3 (*p*=0.008 and *p* < 0.001). Consumption of fibrinogen was higher in the control group on both POD1 (*p*=0.013) and POD3 (*p* < 0.001). Length of hospital stay was lower in the TXA group (5.42 ± 1.21 vs. 6.25 ± 1.49, *p* < 0.001). The transfusion rate and perioperative complications were not significantly different between the two groups.

**Conclusion:**

Administration of TXA is not only effective in reducing perioperative blood loss and length of hospital stay but also exerts an anti-inflammatory effect following CAS-TKA without causing major complications.

## 1. Introduction

Total knee arthroplasty (TKA) is a sophisticated surgery with promising outcomes, owing to advanced surgical techniques and implant designs. However, perioperative complications still occur, some of which are related to substantial blood loss after TKA. On average, blood loss is around 1000–1500 ml after conventional knee arthroplasty, which ultimately results in a blood transfusion rate of 4–17.5% [1–3]. Moreover, transfusions are associated with transfusion-related reactions and infection and have also been identified as a risk factor for circulatory volume overload and periprosthetic joint infection [4–6].

Surgical trauma has been proposed to be one of the major factors related to postoperative blood loss through activation of the fibrinolysis reaction. A pneumatic tourniquet is used in order to create a bloodless surgical field during TKA, which further augments the fibrinolysis reaction, especially after release of the tourniquet [7, 8]. Several approaches have been used to reduce the postoperative blood loss and transfusion rate after TKA, including controlled hypotension, changing the postoperative knee position [9], using a hemostatic agent [10] and employing computer-assisted surgery (CAS) [11]. In comparison to conventional TKA, in which an intramedullary guide is employed to ensure a correct distal femur cut, CAS-TKA, as a way of minimizing surgical trauma, navigates the distal femur cut without breeching the femoral canal, thereby mitigating the release of systemic emboli and reducing blood loss [12, 13].

Tranexamic acid (TXA), as an antifibrinolytic agent, is a synthetic lysine derivative that reduces blood loss through reversible competition for the lysine-binding site on the plasminogen with fibrin. This further inhibits activation of the plasminogen and prevents degradation of fibrin by the plasmin [7]. Therefore, TXA is not only effective in inhibiting the systemic fibrinolysis reaction after surgical trauma but also inhibits the local fibrinolytic activity caused by the use of a tourniquet. In recent decades, growing evidence has demonstrated the beneficial effects of TXA in reducing blood loss and the transfusion rate in several orthopedic surgeries. In the literature, it has been reported that the application of TXA in conventional TKA lowers the blood loss by 300–500 ml on average, resulting in a reduction in the transfusion rate from 4.3% to 0.4% [14, 15]. Moreover, several studies have demonstrated that TXA not only minimizes blood loss but also plays a role in the inflammatory response [16, 17].

Although the effects of TXA and CAS-TKA in reducing blood loss are demonstrable, the additional benefits of using TXA alongside CAS-TKA have not been established in detail.

Therefore, we conducted a comparative study to evaluate the efficacy of TXA in reducing blood loss and the inflammatory response after CAS-TKA.

## 2. Methods

### 2.1. Patients

This retrospective study was conducted using prospectively collected data of 249 patients between Jan 2013 and Oct 2017. The study was approved by our Institutional Review Board. All patients who underwent primary unilateral TKA with computer-assisted surgery by a single surgeon for end-stage osteoarthritis were included. Patients who had secondary osteoarthritis (rheumatoid arthritis, posttraumatic osteoarthritis, or infected arthritis), coagulopathy, a history of venous thromboembolism disease (VTED), a history of ischemic heart disease, a history of stroke, or an allergy to TXA were excluded. Finally, a total of 222 patients were included. The TXA protocol has been used in our hospital since Oct 2015; therefore, the study included 119 patients who underwent CAS-TKA without administration of TXA (control group) and 103 patients who were administered TXA at a dosage of 20 mg/kg intravenously 15 minutes before deflation of the tourniquet (TXA group). Demographic data including age, BMI, gender, baseline hemoglobin (Hb), hematocrit (Hct), C-reactive protein (CRP), D-dimer, fibrinogen, and platelet levels were collected. All patients were subjected to a routine postoperative protocol of blood tests on postoperative days (PODs) 1 and 3 to measure the levels of Hb, Hct, CRP, D-dimer, fibrinogen, and platelet. The transfusion criterion was set at Hb < 8 g/dL or 8–10 g/dL with the presence of symptoms such as dizziness, tachypnea, or hypotension. Prophylactic antibiotic cefazolin (1 g) was administered intravenously preoperatively and every 8 hours postoperatively for 3 doses.

### 2.2. Surgical Technique

CAS-TKA was performed in all the patients under general anesthesia using an infrared-based navigation system (Vector Vision; BrainLAB, Heimstetten, Germany). This optical tracking system detects marker spheres under the infrared camera. Two reference arrays outside the wound were fixed to the distal femur (4 mm pins) and proximal tibia (3 mm pins) using two bicortical half-pins that could be tracked by the infrared camera. The surgery was carried out using the midvastus approach through a midline skin incision under a 280 mmHg pneumatic tourniquet. The hip joint center, distal femur and proximal tibia condyle articular surface, and medial and lateral malleolus of the ankle were mapped and registered in sequence. The femur first gap-based program was used in CAS-TKA. The anterior cortex of the distal femur was chosen as the reference for the femur component, and the transepicondylar axis, Whiteside's line, and posterior condylar line were references for rotation. After registration, the size and position of the femur component were determined by the navigation system. The distal femur and posterior condyle cut were then navigated without breeching of the femoral canal.

The tibia cutting level, angle, and slope were determined using the extramedullary guide and navigation system. The rotation of the tibia was adapted to the femur component and the position of tibia tuberosity. Accurate bone cutting was carried out under navigation. The patella was left without resurfacing.

After bone cutting, both the femur and tibia components (LPS-Flex system, Nexgen; Zimmer, Warsaw, USA) were implanted with cement fixation. A 1/8-inch Hemovac was maintained for 24 hours. The tourniquet was released after wound closure and application of dressings.

### 2.3. Outcome Measurements

The primary outcomes included the levels of Hb, Hct, CRP, D-dimer, fibrinogen, platelet, and the reduction in Hb on POD1 and POD3. The blood volume was estimated via the Nadler formula [18]. The estimated total blood loss (ETBL) was then calculated using the hemoglobin balanced formula [19]. Secondary outcomes of transfusion rate and 90-day perioperative complications were recorded, including superficial wound infection, deep infection, VTED, gastrointestinal bleeding, stroke, myocardial infarction, heart failure, 90-day reoperation, and 30-day readmission.

### 2.4. Statistical Analysis

The collected data are presented as means ± standard deviations for continuous variables and percentages for discrete variables with descriptive statistical analysis. The Kolmogorov–Smirnov test was used to test the normality of the data. The Mann–Whitney *U* test was used for comparison of continuous variables between groups. The chi-square test was employed to compare categorical variables. A *p* value of less than 0.05 was considered significant. All statistical analyses were performed using SPSS software V.21 (SPSS Inc. Chicago, IL, USA).

## 3. Results

### 3.1. Patient Demographic Characteristics

From Jan 2013 to Oct 2017, a total of 222 patients were enrolled in this study, including 119 patients in the control group and 103 patients in the TXA group, with a mean age of 69.63 ± 7.73 and 69.63 ± 7.51 years, respectively. There were no significant differences between groups regarding age, gender, BMI, preoperative Hct, CRP, D-dimer, fibrinogen, and platelet levels; however, the Hb level was lower in the TXA group than the control group (12.82 ± 1.27 vs. 13.19 ± 1.34, *p*=0.039). The tourniquet duration (min) was 100.47 ± 17.36 min in the control group and 98.50 ± 20.84 min in the TXA group (*p*=0.517). Length of hospital stay was shorter in the TXA group (5.42 ± 1.21 vs. 6.25 ± 1.49, *p* < 0.001). The demographic data are summarized in Table 1.

### 3.2. Calculated Blood Loss and Hb Loss

**Figure 1:**
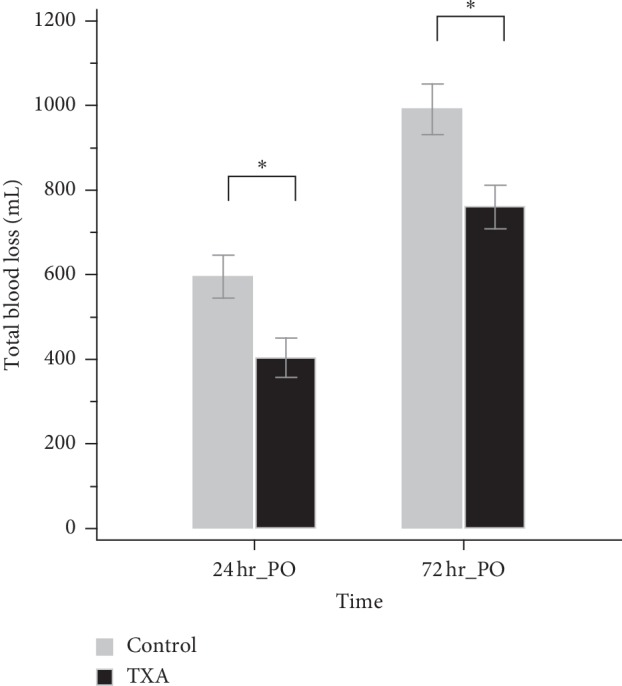
Estimated total blood loss. 24 hr PO: 24 hours postoperatively; 72 hr PO: 72 hours postoperatively; ^*∗*^*p* < 0.05.

The mean ETBL was significantly lower in the TXA group than the control group on both POD1 (404.34 ± 234.77 ml vs. 595.47 ± 279.04 ml, *p* < 0.001) and POD3 (761.39 ± 260.88 ml vs. 987.79 ± 326.58 ml, *p* < 0.001), with an average reduction in blood loss of 22.9% after TXA administration (Figure 1). The reduction in the Hb level was greater in the control group than in the TXA group on POD1 (2.10 ± 0.99 vs. 1.34 ± 0.88, *p* < 0.001) and POD3 (3.48 ± 1.13 vs. 2.51 ± 0.89, *p* < 0.001), which resulted in a lower level of Hb in the control group (POD1: 11.08 ± 1.31 vs. 11.48 ± 1.16, *p*=0.011; POD3: 9.71 ± 1.29 vs. 10.32 ± 1.15, *p* < 0.001), as shown in Table 2.

### 3.3. CRP, D-Dimer, Fibrinogen, and Platelet

**Figure 2:**
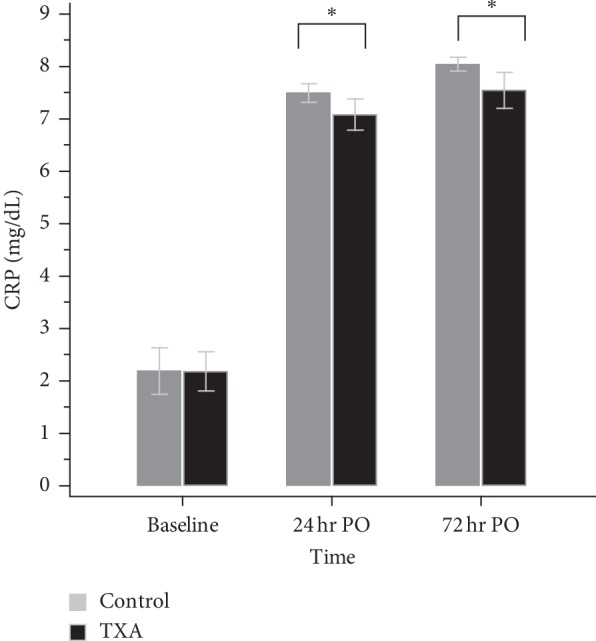
The level of CRP. 24 hr PO: 24 hours postoperatively; TXA group *N* = 100; control group *N* = 107. 72 hr PO: 72 hours postoperatively; TXA group *N* = 83; control group *N* = 79; ^*∗*^*p* < 0.05.

**Figure 3:**
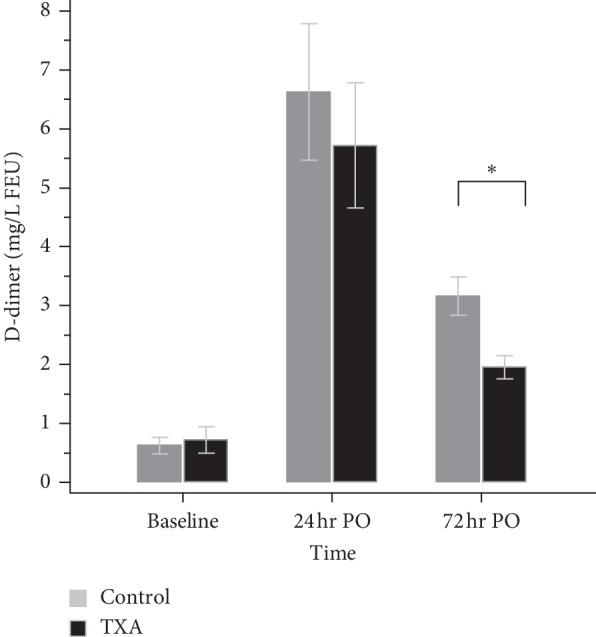
The level of D-dimer. 24 hr PO: 24 hours postoperatively; 72 hr PO: 72 hours postoperatively; ^*∗*^*p* < 0.05.

**Figure 4:**
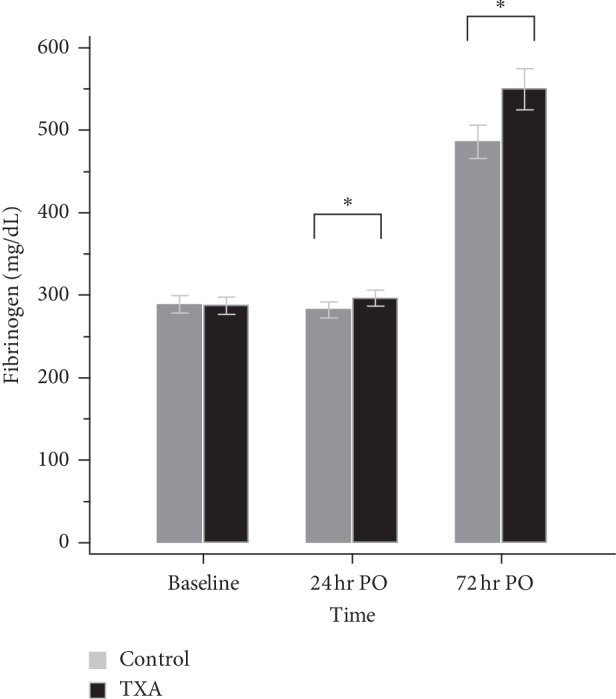
The level of fibrinogen. 24 hr PO: 24 hours postoperatively; 72 hr PO: 72 hours postoperatively; ^*∗*^*p* < 0.05.

The TXA group demonstrated a lower level of CRP than the control group on both POD1 (*N* = 100, 7.08 ± 1.50 vs. *N* = 107, 7.49 ± 0.92, *p*=0.02) and POD3 (*N* = 83, 7.55 ± 1.55 vs. *N* = 79, 8.04 ± 0.60, *p*=0.008) (Figure 2) despite there are missing data of CRP. The mean serum level of D-dimer was similar between groups on POD1 (*p*=0.307) but lower in the TXA group on POD3 (1.95 ± 0.99, vs. 3.16 ± 1.79, *p* < 0.001) (Figure 3). Regarding the fibrinogen, the consumption was higher in the control group which resulted in a lower level than the TXA group on both POD1 (282.29 ± 51.68 vs. 296.48 ± 49.01, *p*=0.013) and POD3 (485.74 ± 111.84 vs. 549.82 ± 128.34, *p* < 0.001) (Figure 4). There is no significant difference in platelet levels between two groups on POD3, as shown in Table 2.

### 3.4. Transfusion and Complications

The control and TXA groups did not differ significantly regarding the transfusion rate, with 2 patients in the control group and no patients in the TXA group undergoing transfusions (1.7% vs. 0%, respectively, *p*=0.501). Similar results were observed with respect to the 90-day perioperative complications in the two groups. In the control group, 1 patient (0.8%) sustained a superficial wound infection, 1 (0.8%) contracted a deep infection, 1 (0.8%) suffered GI bleeding, 1 (0.8%) required a reoperation within 90 days for acute periprosthetic joint infection, and 1 (0.8%) was readmitted within 30 days for transient change of consciousness. In comparison, in the TXA group, 1 patient (1%) sustained a superficial wound infection and 1 (1%) was readmitted within 30 days for postoperative swelling and hematoma (*p*=1.000, respectively), as shown in Table 3.

## 4. Discussion

TXA has been commonly used in recent decades, owing to its high efficacy in reducing perioperative blood loss and the blood transfusion rate after conventional TKA and its low cost [14, 15]. In the present study, we demonstrated an average reduction in blood loss of 22.9% after TXA administration during CAS-TKA (761.39 ± 260.88 ml vs. 987.79 ± 326.58 ml, *p* < 0.001). In addition to the reduction in blood loss, a lower level of D-dimer (*p* < 0.001), CRP (*p* < 0.008), and a lesser consumption of fibrinogen (*p* < 0.001) were observed in the TXA group.

In response to surgical stress, a cascade of inflammatory and coagulatory reaction is activated, which results in a shift of the hemostatic mechanism towards a hyperfibrinolysis status. Therefore, the use of TXA as an antifibrinolytic agent is effective in controlling postoperative bleeding related to fibrinolysis [20]. Although CAS-TKA can reduce blood loss by 100–400 ml in comparison to conventional TKA [12], the added benefit of using TXA in CAS-TKA has not been well established. Therefore, the goal of the current study was to evaluate the efficacy and safety of TXA in CAS-TKA, with emphasis on the perioperative total blood loss and additional markers, including the levels of CRP, D-dimer, and fibrinogen.

The proposed mechanism of blood loss after TKA includes direct loss via surgical trauma and extravasation through vessels after release of the tourniquet. Existing evidence has demonstrated that TXA can not only reduce the drainage volume by 50%, which is mainly affected by surgical trauma [7, 21], but only contributes to a 30% reduction in total blood loss after TKA, which consists of hidden blood loss and direct loss [19]. Hidden blood loss occurs mainly at the time of vessel extravasation after release of the tourniquet prior to activation of the fibrinolysis reaction. As reported by Good et al., TXA is less effective in controlling hidden blood loss [19]. In CAS-TKA, direct blood loss through surgical trauma is already mitigated by avoidance of violation of the femoral canal, and therefore, TXA may be of an inferior efficacy in CAS-TKA in comparison to administration during conventional TKA. According to a review of the literature, TXA reduces blood loss by 30–50% in conventional TKA [22, 23]. In this study, we demonstrated a reduction in total blood loss of 22.9% after administration of TXA in CAS-TKA, which represented a lower effectiveness than when used in conventional TKA, but was in line with current literature regarding navigated arthroplasty. Song et al. [24] reported a 23.1% reduction in drain loss and a 15.6% reduction in total blood loss when TXA was administered during CAS-TKA, while McConnell et al. [25] showed a reduction in total blood loss of 22.8% following navigated TKA, with a loss of 1149 ml in patients without TXA and 886 ml in patients administered with TXA. Moreover, numerous studies have shown that TXA is of benefit not only in reducing blood loss but also in reducing the transfusion rate. Yang et al. conducted a meta-analysis on the effectiveness of TXA, the results of which indicated a reduction in blood loss of 504 ml and a decrease in the number of units transfused per patient of 1.43 units after conventional TKA [14]. Regarding the impact of CAS-TKA on transfusions, Licini and Meneghini [13] reported a significant reduction in total blood loss, with a loss of 1327 ml in conventional TKA and 925 ml in CAS-TKA, with similar transfusion rates in both groups. A recent meta-analysis which included 12 studies also demonstrated an average reduction in a total blood loss of 185 ml when employing the CAS approach, with no significant difference in the transfusion rate [12]. In the current study, we revealed a reduction in total blood loss from 988 ml to 761 ml with administration of TXA, with transfusion rates of 1.7% and 0% in the control group and TXA group, respectively, which did not represent a statistically significant difference. As the use of CAS in TKA already reduces blood loss by 10–30% [12], our results suggest that TXA has a synergistic effect when applied in CAS-TKA.

As a substrate for fibrin formation, fibrinogen is consumed to make a clot in response to the fibrinolytic reaction after surgical trauma. A related issue concerns the effects of TXA on fibrinogen kinetics in adult spinal deformity surgery. Pong et al. [26] demonstrated that the reduction of fibrinogen was lessened in all groups administered TXA, indicating a lower consumption of the substrate under the antifibrinolysis effects of TXA. In the current study, we observed a higher fibrinogen level in the TXA group than in the control group on both POD1 and POD3. This finding seems to be indicative of the fact that TXA exerts an antifibrinolytic effect in CAS-TKA.

The plasminogen not only undergoes fibrinolysis though binding with fibrin on its lysine-binding site but also binds with receptors on some inflammatory cells, such as monocytes, macrophages, neutrophils, endothelial cells, and platelets [27]. TXA, a lysine derivative, reversibly competes for the lysine-binding site on the plasminogen. Therefore, despite its blood-saving properties, TXA might exert an anti-inflammatory effect; this remains controversial in the application of TKA. In a pilot study conducted by Grant et al., it was observed that TXA significantly increased the expression of MCP-1, TNF-*α*, and IL-6 after bone cutting as compared with non-TXA patients [28]. In contrast, Lei et al. [17] and Xie et al. [16] demonstrated a lower level of IL-6 and CRP after administration of TXA in patients undergoing total hip arthroplasty as compared with patients without TXA. Moreover, they found that the higher the number of boluses of TXA, the lower the level of IL-6 and CRP. In CAS-TKA, Kuo et al. [29] highlighted lower levels of systemic and local inflammatory markers than were observed in conventional TKA. According to the existing literature, inflammatory markers not only predict major complications, such as pneumonia, infection, or cardiovascular events, after orthopedic surgeries, but are also correlated with functional recovery after total joint arthroplasties [30–32]. The serum level of D-dimer, a terminal fibrin degradation product, could reflect the fibrinolysis reaction, which peaks at 6 hours after TKA and remains for 18 hours [33]. In an *in vitro* study, Robson et al. [34] observed that exposure of human monocytes to D-dimer boosts the secretion of IL‐1*β* and IL-6, which might indicate that elevation of the D-dimer level plays a significant role in the release of proinflammatory cytokines. In a prospective study, Shahi et al. reported the value of D-dimer not only for diagnosis of periprosthetic joint infection but also for determining the presence of current infection [35]. Furthermore, Lei et al. not only demonstrated a lower serum level of IL-6 following multiple boluses of TXA but also a lower level of D-dimer in comparison with patients without TXA or with one dose of TXA [17]. As Bergin et al. [36] reported, they demonstrated postoperative blood loss was associated with inflammation markers like CRP and IL-6 in patients following THA. In this study, we observed lower levels of CRP and D-dimer in the TXA group than in the control group, which was in line with the results reported in the literature.

Although benefits of TXA in major orthopedic surgeries have been reported since the 1960s, safety concerns remain, owing to its interference in the coagulation cascade. Particular concerns such as the risk of pulmonary embolism, VTED, myocardial infarction, or cerebrovascular events might defer the usage of TXA in joint arthroplasty. With regard to TXA-related complications, several meta-analyses have shown that TXA does not increase perioperative complications in total joint arthroplasty, especially VTED and pulmonary embolism. Our data not only suggested that TXA administration did not increase major complications in CAS-TKA but also demonstrated shorter length of hospital stay; however, we can only demonstrate the safety of TXA administration in particular patients, owing to the exclusion of high-risk patients at the beginning of the study.

Our study had some limitations. First, the study was conducted in a retrospective manner. Second, even the power is enough to demonstrate significant difference on CRP, and few missing data of them may exert bias. Third, the anti-inflammatory effect of TXA was demonstrated through CRP and D-dimer. A lack of inflammatory markers such as IL-6, IL-1, and TNF-*α* may not reflect the inflammatory response. Furthermore, case-control studies of inflammatory markers to examine the effects of TXA in CAS-TKA are necessary.

## 5. Conclusion

The administration of TXA is not only effective in reducing perioperative blood loss and length of hospital stay but also exerts an anti-inflammatory effect during CAS-TKA without causing major complications.

## Figures and Tables

**Table 1:** Baseline demographic data.

	TXA, *N* = 103	Control, *N* = 119	*p* value
Age	69.63 ± 7.51	69.63 ± 7.73	0.999
Gender (M/F)	23/80	18/101	0.150
BMI	28.16 ± 4.59	27.60 ± 3.74	0.312
Pre-op Hb (g/dL)	12.82 ± 1.27	13.19 ± 1.34	**0.039**
Pre-op Hct (%)	39.11 ± 3.49	39.66 ± 3.69	0.247
Pre-op CRP (mg/dL)	2.18 ± 1.92	2.19 ± 2.37	0.978
Pre-op D-dimer (mg/L)	0.72 ± 1.12	0.63 ± 0.79	0.847
Pre-op fibrinogen (mg/dL)	287.38 ± 51.82	288.87 ± 58.51	0.989
Pre-op platelet (1000/*μ*L)	225.71 ± 51.07	234.82 ± 56.02	0.161
Tourniquet time (min)	98.50 ± 20.84	100.47 ± 17.36	0.517
Length of hospital stay (day)	5.42 ± 1.21	6.25 ± 1.49	<0.001

Pre-op: preoperative; Hb: hemoglobin; Hct: hematocrit.

**Table 2:** Postoperative parameters.

	TXA, *N* = 103	Control, *N* = 119	*p* value
POD1			
Hb (g/dL)	11.48 ± 1.16	11.08 ± 1.31	0.011
Hct (%)	34.67 ± 3.33	33.00 ± 3.76	<0.001
D-dimer (mg/L)	5.70 ± 5.39	6.63 ± 6.38	0.307
Hb drop (g/dL)	1.34 ± 0.88	2.10 ± 0.99	<0.001
Fibrinogen (mg/dL)	296.48 ± 49.01	282.29 ± 51.68	0.013
POD3			
Hb (g/dL)	10.32 ± 1.15	9.71 ± 1.29	<0.001
Hct (%)	31.15 ± 3.45	29.10 ± 3.81	<0.001
D-dimer (mg/L)	1.95 ± 0.99	3.16 ± 1.79	<0.001
Hb drop (g/dL)	2.51 ± 0.89	3.48 ± 1.13	<0.001
Fibrinogen (mg/dL)	549.82 ± 128.34	485.74 ± 111.84	<0.001
Platelet (1000/*μ*L)	176.24 ± 38.40	181.08 ± 47.11	0.587
*POD1 ETBL (ml)*	404.34 ± 234.77	595.47 ± 279.04	<0.001
*POD2 ETBL (ml)*	761.39 ± 260.88	987.79 ± 326.58	<0.001
*Transfusion rate (%)*	0	2 (1.7%)	0.501

Hb: hemoglobin; Hct: hematocrit; ETBL: estimate total blood loss.

**Table 3:** Complications within 90 days.

	TXA group, *N* = 102	Control group, *N* = 119	*p* value
Superficial wound infection	1 (1%)	1 (0.8%)	1.000
Deep infection	0	1 (0.8%)	1.000
Venous thromboembolism	0	0	
GI bleeding	0	1 (0.8%)	1.000
Stroke	0	0	
Myocardial infarction	0	0	
Heart failure	0	0	
90-day reoperation	0	1 (0.8%)	1.000
30-day readmission	1 (1%)	2 (1.7%)	1.000

## Data Availability

All data supporting the results can be found in the manuscript. The raw data can be provided on request.
